# Risks and Challenges of Arboviral Diseases in Sudan: The Urgent Need for Actions

**DOI:** 10.3390/v12010081

**Published:** 2020-01-09

**Authors:** Ayman Ahmed, Isabelle Dietrich, A. Desiree LaBeaud, Steve W. Lindsay, Ahmed Musa, Scott C. Weaver

**Affiliations:** 1 Institute of Endemic Diseases, University of Khartoum, Khartoum 11111, Sudan; 2 Institute for Human Infections and Immunity, Department of Microbiology and Immunology, University of Texas Medical Branch, Galveston, TX 77755, USA; 3 World Reference Center for Emerging Viruses and Arboviruses, University of Texas Medical Branch, Galveston, TX 77755, USA; 4 The Pirbright Institute, Surrey GU24 0NF, UK; 5 School of Medicine, Stanford University, Stanford, CA 94305, USA; 6 Department of Biosciences, Durham University, Durham DH1 3LE, UK

**Keywords:** arthropod-borne viruses, arboviruses, mosquito-borne viruses, sandfly-borne viruses, tick-borne viruses, arboviral diseases, emergence, epidemics, outbreaks, Sudan

## Abstract

The risk of emergence and/or re-emergence of arthropod-borne viral (arboviral) infections is rapidly growing worldwide, particularly in Africa. The burden of arboviral infections and diseases is not well scrutinized because of the inefficient surveillance systems in endemic countries. Furthermore, the health systems are fully occupied by the burden of other co-existing febrile illnesses, especially malaria. In this review we summarize the epidemiology and risk factors associated with the major human arboviral diseases and highlight the gap in knowledge, research, and control in Sudan. Published data in English up to March 2019 were reviewed and are discussed to identify the risks and challenges for the control of arboviruses in the country. In addition, the lack of suitable diagnostic tools such as viral genome sequencing, and the urgent need for establishing a genomic database of the circulating viruses and potential sources of entry are discussed. Moreover, the research and healthcare gaps and global health threats are analyzed, and suggestions for developing strategic health policy for the prevention and control of arboviruses with focus on building the local diagnostic and research capacity and establishing an early warning surveillance system for the early detection and containment of arboviral epidemics are offered.

## 1. Introduction

Arthropod-borne viruses (arboviruses) have received much global attention recently due to the Zika virus epidemic in the Americas. The major human arboviruses belong to the Togaviridae, Flaviviridae, or Peribunyaviridae families [1,2]. These viruses cause serious diseases including dengue fever, yellow fever, Rift Valley fever, chikungunya, and various arboviral encephalitides. The risk of arboviral diseases is on the increase and their geographical distribution has expanded [2]. Nevertheless, little is known about the epidemiology, distribution, and dynamics of arboviruses in Africa [1]. Due to limitations in the capacity of the health systems, particularly at the level of diagnosis of infectious diseases, many cases of arboviral diseases are misdiagnosed as malaria or any other similar febrile illness. This, in turn, not only leads to an overestimation of malaria episodes, but also to an underestimation of arboviral diseases and their socio-economic impacts [3,4,5,6].

Several risk factors contribute to the emergence and re-emergence of arboviral diseases. These include climate change, international travel and trade, increased contact of humans and wild or domesticated animals, an explosion of the human population, and unplanned urbanization [7,8,9,10,11]. The limited evidence and published data about the risk of arboviruses in Africa restrains the ability of health systems to develop strategic interventions to control arboviral diseases [4]. Most arboviruses are zoonotic with frequent spillover and spillback between human and animal populations [2,12]. Interestingly, Sudan is located at a crossroad of international human travel routes, animal exportation and transportation, and seasonal bird migration routes, which predisposes the Sudanese population to arbovirus contact [2,13,14]. Considering these factors, understanding the epidemiology of arboviruses in Sudan is of global concern. Therefore, in this review, we aim to shed light on the risk of arboviruses in Sudan and highlight the main gaps in knowledge, research, and control of the major human and veterinary arboviral diseases as well as the main challenges to develop a strategic plan for control.

## 2. Epidemiology of the Major Arboviral Diseases in Sudan

### 2.1. Mosquito-Borne Viral Diseases

#### 2.1.1. Yellow Fever Virus

**Figure 1:**
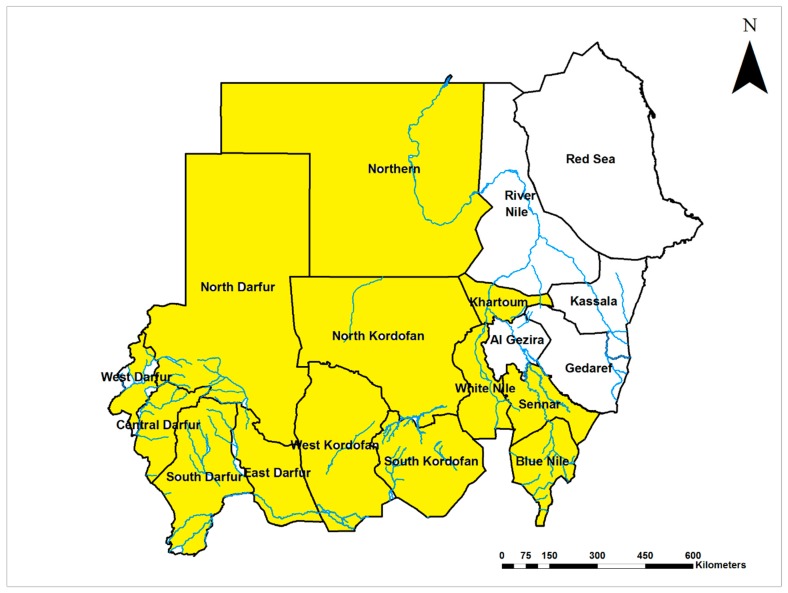
Map of Sudan showing the prevalence of Yellow fever virus infections (Yellow).

Yellow fever virus (YFV; Flaviviridae) is endemic in Sudan and infections occur throughout the country (Figure 1). Early studies suggested that yellow fever (YF) has been endemic in the middle and western regions of the country since the 1800s [15]. The world’s worst recorded outbreak of YF, with more than 40,000 cases and 1500 deaths, was reported from 1940 to 1941. YFV was detected in humans, non-human primates, wild and domestic animals in 1941, 1952, and 1953 [15,16,17,18,19,20,21,22,23,24,25,26]. Soon thereafter, a second epidemic of lesser magnitude occurred in 1959 in the southern region of Sudan, with 114 cases and a 77% case fatality rate identified in the area, despite the high vaccine coverage (70%) [27]. A survey in 1973 confirmed the active circulation of YFV in the area and indicated a high prevalence of prior exposure (95%) among the nonvaccinated population of central Sudan [28]. YFV was detected in the Northern Province of Sudan in 1989 with high infection rates (39%) during an epidemic of febrile illness following heavy rainfall and flooding [6]. Other outbreaks took place in southern and western Sudan in 2005 and 2012–13, respectively. The low vaccine coverage prior to the 2005 outbreak and heavy rain were associated with the re-emergence of YF in western Sudan [29]. The 2012 epidemic, considered the worst in Africa in almost quarter of a century (1992–2016, only superseded by the Angola epidemic in 2016) with more than 800 confirmed cases and about 200 related deaths, primarily affected refugees living in camps. Case fatality rates ranged between 20% and 32% [29,30,31,32,33,34,35]. Unfortunately, the lack of a local arbovirology reference laboratory not only delayed the official confirmation of the outbreak but left the epidemic to grow exponentially with the local community unaware of the public health risk. Further, it allowed the virus to spill over into the neighboring country of Chad [32].

#### 2.1.2. Dengue Virus

**Figure 2:**
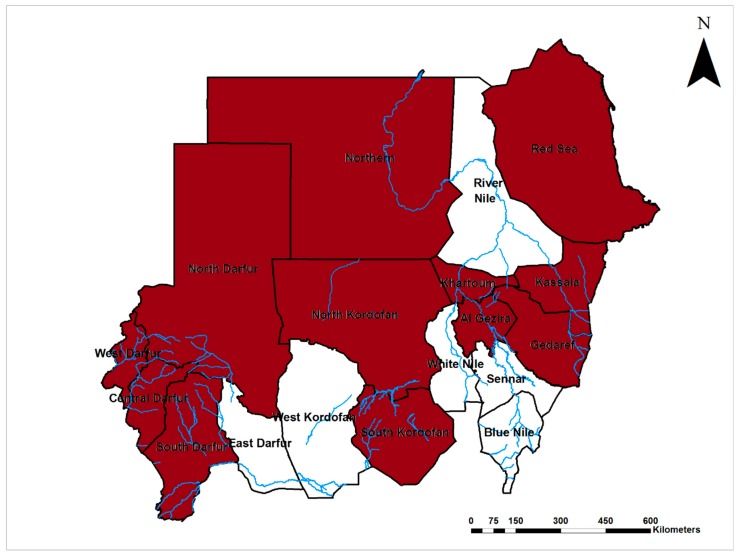
Map of Sudan showing the prevalence of dengue fever virus infections (Brown).

Dengue virus (DENV; Flaviviridae) is endemic in Sudan and its distribution was confined to the coastal and subcoastal regions of the country, where the disease was described for the first time by Saigh in 1906 and Balfour in 1907 on the Red Sea coast, Port Sudan city [36,37,38,39]. Recently, DENV has been emerging throughout the country (Figure 2). Indeed, most dengue outbreaks in the Middle East and North Africa were reported from Sudan [40,41]. Sudan was the first country in Africa to report DENV-1 in 1984; DENV-2 was isolated from patients admitted to a hospital in East Sudan in 1984, and reported from Kassala state for the first time during an outbreak in 2016/17 [42,43]. DENV infections were reported from the population of east, north, south, and central Sudan with infection rates ranging between 7–25%, and these infections were associated with epidemics of febrile illness, following heavy rain and flooding [5,6,35]. Studies of DENV infection among pregnant women in East Sudan showed that they commonly develop severe forms of disease leading to poor maternal and perinatal outcomes, including 22% maternal death, 24% low birth weight, and 18% preterm delivery [3,44,45]. In 2012, a relatively high prevalence of dengue and measles co-infection (17%) in addition to 20% and 12% independent measles and dengue infections, respectively, have placed a great diagnostic challenge on the resource-limited health system. All of these infections were initially diagnosed as measles, indicating the diagnostic challenge in endemic areas of east Sudan [46]. An epidemic of dengue hemorrhagic fever (DHF) with 312 cases occurred among children under 15 years of age, and about 12% of them manifested with dengue shock syndrome (DSS). DENV-3 was isolated from patients during the outbreaks, and a 4% case fatality rate was reported in Port Sudan city, East Sudan in 2005 [47]. Outbreaks of dengue fever occurred frequently in the coastal and subcoastal areas of the country. Another major epidemic caused by DENV-3 occurred in 2010 with incidence up to 72%. About two thirds of cases presented with DHF and DSS, and more than 4000 cases were detected [41,48,49,50]. Entomological surveillance showed that *Aedes aegypti*, notorious as the major vector of this virus in urban areas, was the predominant mosquito species in the area. Later, in 2015, a novel outbreak of dengue fever occurred among refugees in the Darfur area, west Sudan, in which DENV-2 and 3 were co-circulating in the area [51]. This was followed by further outbreak in 2016/17 in east Sudan caused by DENV-2 [42]. Additionally, studies reported a high prevalence (67%) of DENV infections in Sudan and up to 89% in the coastal area. They highlighted that most people in these areas were infected repeatedly, and reported that all DENV (1–4) serotypes are circulating in the area [52,53]. Recently, DENV-1 and 3 emerged and caused an outbreak of dengue fever in the Darfur area, western Sudan [54]. DENV transmission in Sudan is heterogeneous in space and time, and the major risk factors for infection were poverty, lack of mosquito control, sleeping outdoors, lack of basic public services such as water supply, storing drinking water, and geographical location [41,53].

#### 2.1.3. West Nile Virus

**Figure 3:**
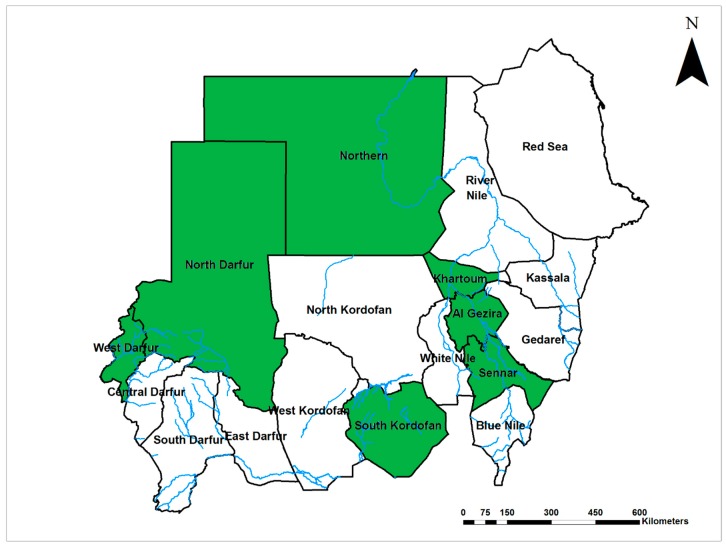
Map of Sudan showing the prevalence of West Nile virus infections (Green).

West Nile virus (WNV, Flaviviridae) is endemic in Sudan with infections reported in different regions of the country since 1942 and 1953–1954 (Figure 3) [6,35,55,56]. Salim and Porterfield suggested that WNV circulates enzootically in central Sudan, and this was confirmed in 1981 by Omer et al. [28,57]. Country-wide epidemics of WNV infection were reported in 1994, 1996, and 1999, with infection rates up to 59%, associated with heavy rainfall and flooding [5,6,58]. An outbreak of WNV infection in 2002, southern Sudan, was accompanied by severe neurological manifestations and the development of encephalitis among 31 children under 12 years of age [59]. WNV infections have been recently identified in Darfur region, West Sudan in 2015 [54]. There is a significant gap in knowledge about the epidemiology and risk factors associated with WNV infection, and local vectors have never been investigated.

#### 2.1.4. Zika Virus

**Figure 4:**
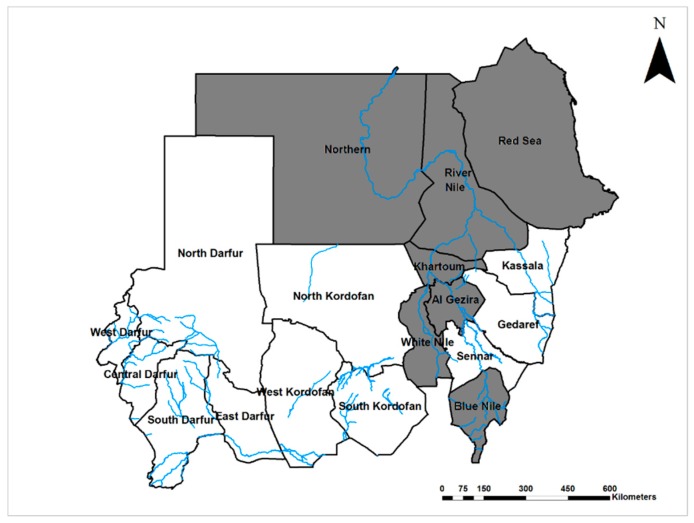
Map of Sudan showing the prevalence of Zika virus infections (Grey).

The first evidence of Zika virus (ZIKV; Flaviviridae) circulation in Sudan was reported in 1981 from El Gezira state, central Sudan [57]. A recent study reported a high rate of exposure to ZIKV with 62.7% of 845 blood samples collected from all regions of the country seropositive by ELISA (although only one was positive by the more specific neutralization test) [60]. Considering the recent association of ZIKV infection in pregnant women with congenital defects in their infants, and the wide distribution of ZIKV in Sudan (present in seven of 18 states of the country (Figure 4)), studying ZIKV and it is epidemiology in Sudan is remarkably limited.

#### 2.1.5. Chikungunya Virus

**Figure 5:**
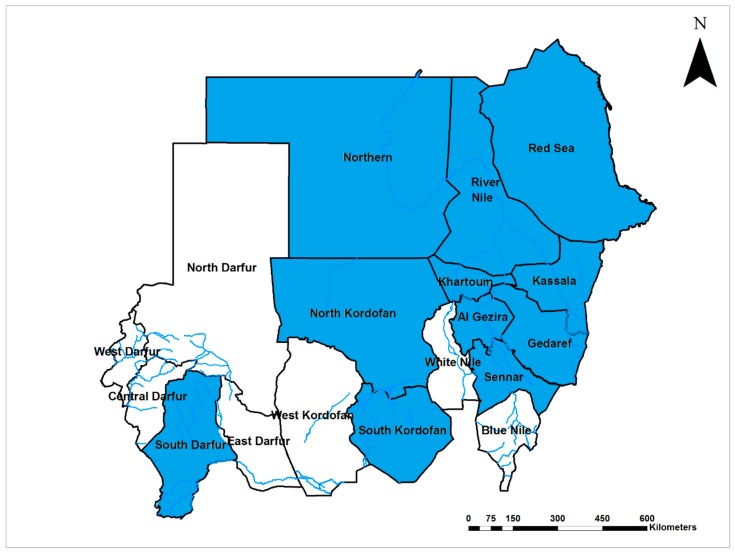
Map of Sudan showing the prevalence of chikungunya virus infections (Blue).

Chikungunya virus (CHIKV; Togaviridae) is endemic with frequent upsurges in Sudan [5,6,28,35,61] and infections documented in seven of the 18 Sudanese states (Figure 5). The first report of the disease in Sudan in 1973 showed that the seroprevalence of CHIKV infection in Central Sudan was 13% [28]. Similar seroprevalences were documented in the central and northern provinces of Sudan, with 10% and 12%, respectively [5,6]. However, studies conducted in the western region of the country documented much higher CHIKV seroprevalence, up to 44% [29,35]. An epidemiological survey in East Sudan using quantitative reverse transcription polymerase chain reaction (qRT-PCR) showed that 24% of pregnant women were infected with CHIKV and the infections were associated with preterm delivery [44]. A wider study across the country reported a relatively low seroprevalence of CHIKV in East and Central Sudan (1.8%) with 100% dengue virus seropositivity [61]. A large-scale outbreak of chikungunya was ongoing in East Sudan between May 2018 and March 2019, with more than 47,000 laboratory-confirmed cases [62,63]. Understandably, during epidemics of febrile illnesses, it is challenging to distinguish arboviral diseases from malaria and other infections due to the lack of laboratory capacity for proper diagnostics [5,6]. Despite the frequent reports of chikungunya from different parts of Sudan, there were no entomological studies investigating the vectors of the disease [61].

#### 2.1.6. O’nyong-Nyong Virus

The circulation of the only established Anopheles-mosquito-borne human arboviral pathogen, o’nyong-nyong virus (ONNV), has been reported in Central Sudan with seroprevalence of 13% among the population of Sennar State [28]. Previous studies suggested a major epidemic, with 2 million human infections, occurred in East African countries, including Sudan, between 1959 and 1962 [64,65]. Unfortunately, there are no recent studies providing up-to-date information about the epidemiology and the associated risk factors for ONNV in Sudan.

#### 2.1.7. Rift Valley Fever Virus

**Figure 6:**
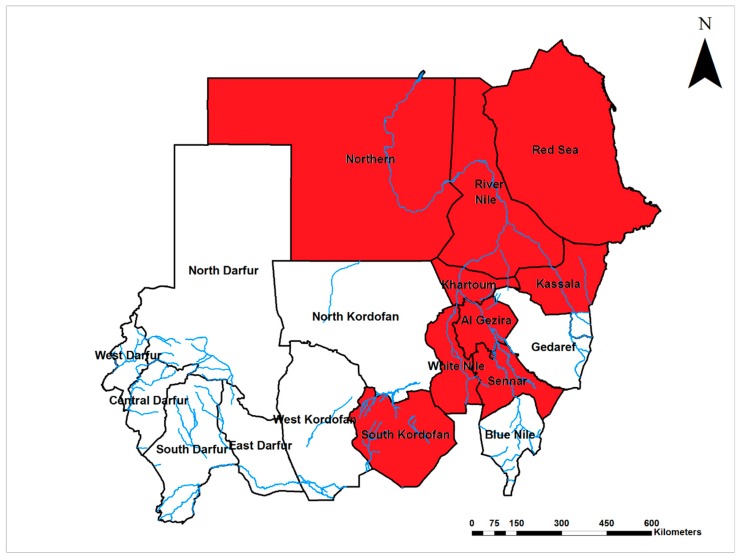
Map of Sudan showing the prevalence of Rift Valley fever virus infections (Red).

Rift Valley fever virus (RVFV; Phenuiviridae) is endemic with frequent outbreaks in Sudan, mainly in the central region and rich Savannah area, with continuous active transmission in both humans and animals [5,6,66,67]. RVFV infections have been reported in 50% (9/18) of Sudanese States (Figure 6). RVFV was first detected in central Sudan in the 1930 s, then reported from southern Sudan in 1959 [55,68]. Several epizootics and epidemics took place in 1973, 1976–7, 1984, 2001, 2007–8, 2010, and 2016 [67,69,70,71,72]. It is hypothesized that RVFV has been introduced into Egypt from Sudan through animal exportation [73]. The circulation of RVFV was reported in central and northern Sudan [5,6,74]. Two major outbreaks occurred in Sudan in 2007 and 2010. In 2007, the outbreak was most devastating, with more than 700 confirmed human cases and a case fatality rate of 30.8%; estimated infections were up to 75,000, resulting in a great economic loss due to banning of livestock trade. Direct contact with infected animals and the use of unpasteurized or uncooked animal products were major risk factors [66,75,76,77,78,79,80]. RVFV infection was strongly associated with developing renal disease, with renal impairment reported from 60% of the 194 RVF patients admitted to Wad Medani Teaching hospital in 2007 [81]. The limited phylogenetic investigations carried out in the 2007 and 2010 epidemics suggested multiple introductions into Sudan. All of the introduced strains clustered in the Kenya 1 and 2 sublineages, with the possible presence of other lineages or additional virus introduction events [76]. A case of vertical RVFV transmission was reported from the Khartoum teaching hospital [82]. Furthermore, miscarriage in humans was strongly associated with RVF [44]. RVFV was detected in larval and adult stages of several species of mosquitoes (*Ae. aegypti*, *Ae. vittatus*, *Ae. vexans*, *Anopheles arabiensis*, *An. coustani*, and *Culex quinquefasciatus*) during an outbreak of RVFV in Sudan, suggesting vertical transmission, but the relative importance of each species in transmission was not determined [83,84]. A serological survey showed that the circulation of RVFV persists among the animal populations of Khartoum [69]. Human risk factors associated with RVF include close contact with infected animals, and consumption of products from sick animals, heavy rainfall, and the type of soil and land topography [66,75,79,85]. Unfortunately, most animal breeders do not report suspected infections to the veterinary or health authority to avoid the culling of their herds without compensation as a control measure for epizootics [86].

### 2.2. Tick-Borne Viral Diseases

**Figure 7:**
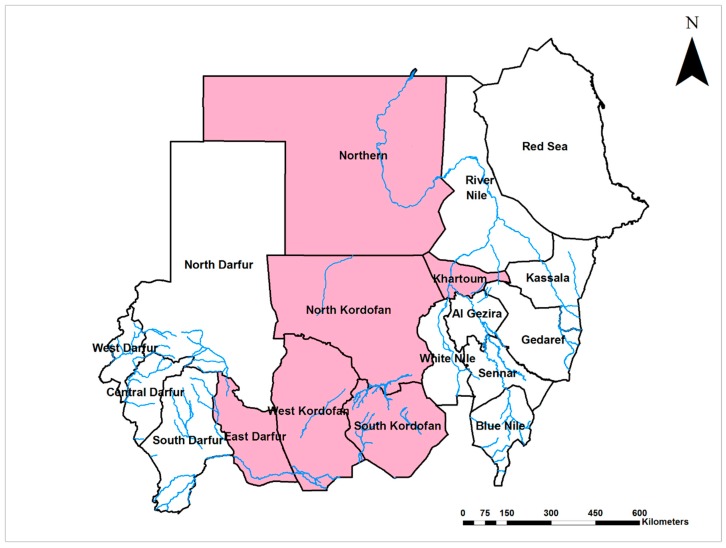
Map of Sudan showing the prevalence of Crimean-Congo hemorrhagic fever virus infections (Pink).

Crimean-Congo hemorrhagic fever virus (CCHFV; Nairoviridae) is endemic in Sudan, and infections have been documented throughout the middle region of the country (Figure 7). The seroprevalence of CCHF in the northern province was 5% in 1989 [6]. Two outbreaks of CCHFV infection occurred in central and southern Sudan in 2008 and 2009 with a 57% case fatality rate. Interestingly, despite the short interval between the two epidemics, they were caused by two different strains of CCHFV, the Mauritanian ArD39554, and one strain belonging to the Group III lineage (strains commonly found across several African countries) [13,87]. Two nosocomial outbreaks were reported among healthcare providers who were attending to CCHF patients in endemic areas in southwestern and central Sudan in 2008 and 2010 [13,88]. The circulation of CCHFV in southwestern and central Sudan has been reported in cattle populations in 2013 and 2015, with a concomitant outbreak of CCHF in the human population in 2013 and 2014 [89,90,91]. CCHFV was detected in various Sudanese domestic animals from different areas of the country, showing that there is active transmission of the CCHFV in most of Sudan, with seroprevalence between 7% and 21% [89,91,92]. Tick infestation, age, area, and animal breeding were risk factors for CCHFV infection among animals. Further, animals following an open grazing system were 27 times more likely to become infected [89,91,92]. CCHFV has been isolated from pools of tick including several species removed from Sudanese sheep exported to Saudi Arabia, but local vectors of CCHFV have not been investigated comprehensively [93]. Detection of CCHFV in ticks infesting sheep imported from Sudan to Saudi Arabia suggested that the diseases might have been introduced from Sudan [94].

### 2.3. Sandfly-Borne Viral Diseases

Arumowot virus (AMTV; Bunyaviridae) is a sandfly (Phlebotomus)-borne virus first isolated from a mosquito, Culex antennatus, from Sudan in 1963 [95]. Several sandfly-borne viruses including Sandfly Fever Sicilian (SFSV) (13–16% seroprevalence), Sandfly Fever Naples (SFNV) (14–33%), Arumowot (1.4–16.7%), SudAn. 754–61 (17–28%), and Karimabad viruses (1–4%) have been detected in sera collected in 1975 from different parts of Sudan [96,97]. In 1988, the seroprevalence of SFSV and SFNV in central Sudan was 54% and 34%, respectively, and 53% and 32% in northern Sudan in 1989 [5,6]. These high infection rates of SFS and SFN were detected during epidemics of febrile illness following heavy rains and flooding [5,6]. Further studies about the burden of these viruses and their distributions are urgently needed.

## 3. Risks and Challenges

The burden of arboviral diseases has devastating socioeconomic and health impacts by causing the loss of human and domesticated animals, and reductions in economic production in endemic countries, including Sudan [4]. Unfortunately, the risk, distribution, and global burden of arboviruses is increasing and is predicted to worsen in the future unless serious actions are taken [2]. Nevertheless, a great challenge during epidemics of arboviral diseases in Sudan is the misclassification and misdiagnosis during the acute phase due to the similarity in signs and symptoms between arboviral diseases and several locally circulating infectious diseases, including measles, malaria, and hepatitis, particularly when the infections are severe [3,5,30,33]. This challenge is not restricted to Sudan, and is a common in other resource-limited countries [33]. Additionally, the lack of well-established public health policies or programs for the prevention and control of arboviral diseases in Sudan leaves the local population and international visitors at greater risk of epidemics and potentially, exportation to new areas. Moreover, this risk is influenced by the delay and/or lack of sharing the data and information of epidemics and health emergencies by the health authorities [98]. In addition, there is an obvious weakness in this area of research due to the limited funds, which are mostly directed toward malaria in malaria-endemic countries [1,5]. There is a great need for strengthening the surveillance system and raising awareness of the health policymakers and healthcare providers about the risk of arboviruses, as emphasized in the World Health Organization’s Global Vector Control Response 2017–2030 [99]. Apparently, as arboviral infections are greatly under-reported or misdiagnosed, this masks the magnitude of the problem from the policymakers and leaves the issue worsening. Furthermore, previous local studies failed to identify the risk factors associated with arboviral infections and epidemics, and such information is crucial for developing strategic prevention and control plans [60,61].

**Figure 8:**
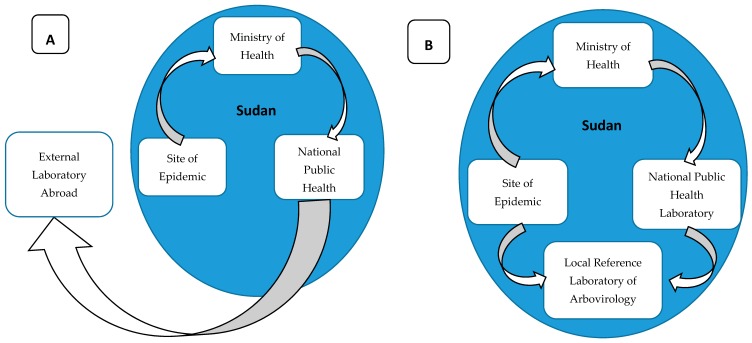
(**A**). Diagram of sample processing for suspected arboviral infections from the field to the confirmation of the arboviral infection. (**B**). Proposed protocol for local, rapid detection and confirmation of arboviral infections based on the establishment of a local, advanced reference laboratory for arbovirology to confirm infections and support the national disease surveillance system.

The major challenge for the prevention, control, and research of arboviruses in Sudan is the limited diagnostic capacity. The Ministry of Health is relying on external laboratories for confirmation of arboviral infections, commonly in Germany or Senegal (Figure 8) [30,31,34], resulting in delays in responses. The lack of advanced diagnostics with high sensitivity creates a great diagnostic challenge for the surveillance system and limits early detection of diseases [3]. This technological gap, in turn, creates a significant limitation in our knowledge, and many unanswered questions about the epidemiology and dynamics of these diseases and their vectors remain. Such information is crucial for developing an effective prevention and control strategy [100,101,102,103,104,105]. In addition to saving many lives, utilizing recent advancements in the biomedical research could help in elucidating the molecular epidemiology, dynamics, and evolution of arboviral diseases, improving our understanding about the transmission of these viruses and reshaping our prevention, control, and containment strategies (Figure 8) [7,12]. There is also a need to develop multisectoral programs for controlling many of these arboviral diseases. This is critically important in Sudan’s towns and cities, where populations of the world’s most important vector of arboviral diseases, Ae. aegypti, are likely to thrive. Genetic and behavioral characterizations of Ae. aegypti populations to determine which represent peridomestic (Ae. aegypti aegypti) versus sylvatic (Ae. aegypti formosus) populations are also needed. In urban areas in particular, trash removal, the provision of reliable piped water, house screening, and infrastructure that does not provide aquatic habitats for this vector is essential for reducing the threat from diseases like chikungunya, dengue, Zika, and yellow fever. As Sudan’s towns and cities continue to grow, there has never been a better time to do this [106].

## 4. Conclusions and Recommendations

Although the risk of arboviral diseases in Sudan is high, it receives little attention, mainly due to limited resources, which heavily undermines building the capacity of healthcare providers and researchers with advanced training. Also, the lack of advanced diagnostic tools is a major challenge as detection of arboviruses typically requires molecular techniques and equipment such as simple polymerase chain reaction (PCR) machines that are available in very limited healthcare facilities. Additionally, the very inadequate funding for research limits the generation of evidence about the epidemiology and dynamics of arboviruses in Sudan; therefore, neither healthcare providers nor health policymakers are aware of these remarkably fast-growing public health threats and the need to develop a multisectoral approach to disease control and prevention. It is critically important to improve surveillance and investigate the circulating strains of these viruses and to establish viral genomic databases as a reference for current and future research. An arbovirology research center in Sudan, which would offer an extraordinary opportunity to advance the research and control of arboviral diseases in the region, is critically needed (Figure 8). This could occur through local and international partnerships to generate evidence about the burden of disease, building the capacity of the local scientists, healthcare providers, and public health policymakers, and raising the awareness of the public and related stakeholders about the risk and need for prevention and control interventions. Drawing global attention to fill gaps in funding, research, and healthcare particularly for the poor and remote communities would likely save many lives and avoid great economic loss.

We highlight the urgent need for raising awareness among healthcare providers, researchers, and policymakers about the issue of arboviral infections, building the capacity of Sudan biomedical laboratories, and increasing the funding for research, particularly to investigate the burden of infectious diseases. Also, improving surveillance, diagnostics, and strengthening the health system overall by adopting a multisectoral approach to control will help in detecting the early emergence of these infections to take action to mediate massive epidemics. Particular attention needs to be paid to reducing the threat of arboviral diseases in the most vulnerable groups in the community, including people living in humanitarian crisis settings, informal settlements, refugees and nomads, and generally, impoverished people throughout Sudan.
